# The *Streptococcus* phage protein paratox is an intrinsically disordered protein

**DOI:** 10.1002/pro.5037

**Published:** 2024-05-27

**Authors:** Iman Asakereh, Nicole R. Rutbeek, Manvir Singh, David Davidson, Gerd Prehna, Mazdak Khajehpour

**Affiliations:** ^1^ Department of Chemistry University of Manitoba Winnipeg Manitoba Canada; ^2^ Department of Microbiology University of Manitoba Winnipeg Manitoba Canada

**Keywords:** bacteriophage, ComR, Hofmeister effects, intrinsically disordered protein, paratox, quorum sensing, *Streptococcus*

## Abstract

The bacteriophage protein paratox (Prx) blocks quorum sensing in its streptococcal host by directly binding the signal receptor and transcription factor ComR. This reduces the ability of *Streptococcus* to uptake environmental DNA and protects phage DNA from damage by recombination. Past work characterizing the Prx:ComR molecular interaction revealed that paratox adopts a well‐ordered globular fold when bound to ComR. However, solution‐state biophysical measurements suggested that Prx may be conformationally dynamic. To address this discrepancy, we investigated the stability and dynamic properties of Prx in solution using circular dichroism, nuclear magnetic resonance, and several fluorescence‐based protein folding assays. Our work shows that under dilute buffer conditions Prx is intrinsically disordered. We also show that the addition of kosmotropic salts or protein stabilizing osmolytes induces Prx folding. However, the solute stabilized fold is different from the conformation Prx adopts when it is bound to ComR. Furthermore, we have characterized Prx folding thermodynamics and folding kinetics through steady‐state fluorescence and stopped flow kinetic measurements. Our results show that Prx is a highly dynamic protein in dilute solution, folding and refolding within the 10 ms timescale. Overall, our results demonstrate that the streptococcal phage protein Prx is an intrinsically disordered protein in a two‐state equilibrium with a solute‐stabilized folded form. Furthermore, the solute‐stabilized fold is likely the predominant form of Prx in a solute‐crowded bacterial cell. Finally, our work suggests that Prx binds and inhibits ComR, and thus quorum sensing in *Streptococcus*, by a combination of conformational selection and induced‐fit binding mechanisms.

## 1 INTRODUCTION

*Streptococcus pyogenes* or Group A Streptococcus (GAS) is a bacterial pathogen that is implicated in several human diseases (Carapetis et al., 2005). GAS can cause mild infections such as impetigo (Chalker et al., 2017) and pharyngitis (Choby, 2009) or deadly conditions such as rheumatic fever (Dooley et al., 2021) and toxic shock syndrome (Mahieu et al., 1995). Central to the pathogenicity of GAS is the presence of bacteriophage encoded toxin genes (Ralph & Carapetis, 2013). These toxin genes are found within the bacteriophage genome and are actively spread among different strains and species of *Streptococcus* through direct phage infection (Penadés et al., 2015). In fact, bacteriophage infection is a primary driving force of clonal diversity in GAS (Beres et al., 2002; Euler et al., 2016).

After infection, lysogenic GAS bacteriophages incorporate their DNA into the GAS genome and persist as stable prophages (McShan et al., 2019). The prophage often encodes a deadly toxin gene near the 3′ end of the prophage. These toxin genes include the superantigen SpeA which is responsible for scarlet fever and toxic shock syndrome (Moon et al., 2006; Norrby‐Teglund & Kotb, 2000). Adjacent to the toxin gene is the *prx* gene that encodes the protein paratox (Prx) (Aziz et al., 2005; Mashburn‐Warren et al., 2018). Furthermore, the toxin gene and *prx* are one genetic cassette that remains intact when the GAS phage exits the lysogenic cycle and self‐excises from the GAS genome (Aziz et al., 2005). The exact purpose of this linkage remains unclear, but current work strongly suggests that the linkage with *prx* is to maintain the genetic integrity of the toxin (Mashburn‐Warren et al., 2018; Rutbeek et al., 2021).

Supporting this hypothesis is the observation that Prx inhibits natural competence in *S. pyogenes* (Mashburn‐Warren et al., 2018). Natural competence (natural transformation) in Streptococcus is a quorum‐sensing regulated process in which bacteria control the expression of a number of genes that encode the machinery for both the acquisition and incorporation of DNA (Johnston et al., 2014). In GAS, natural competence is regulated by the ComRS quorum‐sensing pathway (Mashburn‐Warren et al., 2010, 2012; Neiditch et al., 2017). ComR is a quorum‐sensing receptor and a transcription factor that is part of the RRNPP protein family (Neiditch et al., 2017). ComS is a secreted peptide pheromone that is processed into a mature form termed XIP (SigX inducing peptide) (Mashburn‐Warren et al., 2012). Upon binding to ComR, XIP induces a large conformational change in ComR that results dimerization and the ability to bind to the promoter regions of *comS* (XIP) and *sigX* (Shanker et al., 2016; Talagas et al., 2016). SigX then leads to the expression of late genes required for natural competence (Campbell et al., 1998; Peterson et al., 2000). The expression of Prx is also induced by SigX, resulting in its own ComRS quorum‐sensing regulated expression (Mashburn‐Warren et al., 2012). Prx then acts as a negative regulator of the system by binding to the DNA‐binding‐domain (DBD) of ComR directly blocking interaction with DNA and inhibiting the expression of competence genes (Mashburn‐Warren et al., 2018; Rutbeek et al., 2021).

Prx is a small 60 amino acid protein that has been shown to adopt a globular fold in X‐ray crystal structures alone (Mashburn‐Warren et al., 2018) or when bound to ComR (Rutbeek et al., 2021). However, previous biophysical characterization of Prx using small‐angle X‐ray scattering coupled to size exclusion chromatography (SEC‐SAXS) shows that Prx occupies a larger volume than what is predicted from the folded crystal structure (Rutbeek et al., 2021), even though analytical ultracentrifuge (AUC) data clearly demonstrates that the protein is a monomer in solution (Mashburn‐Warren et al., 2018). The disagreement between the Prx crystal structures and its behavior in solution suggests that Prx is likely dynamic and adopts conformations significantly different from those captured in the crystal structures.

To probe this hypothesis, we have investigated the folding thermodynamics of Prx using circular dichroism (CD), fluorescence, and protein NMR. Our results show that under dilute and physiological solvent conditions, Prx has all the hallmarks of an intrinsically disordered protein (Wright & Dyson, 2015). Furthermore, the addition of salts and molecular crowders induce Prx to adopt a more compact folded form. However, this solute induced Prx fold is different from the conformation that Prx adopts when it is bound to ComR. We demonstrate that the folding of Prx before interaction with ComR can be properly described by a two‐state thermodynamic model (Eliezer, 2009; Uversky, 2014; Zeng et al., 2022) and propose that under physiological conditions Prx should be assumed to be at dynamic equilibrium between an intrinsically disordered conformational ensemble and a solute‐stabilized fold. This suggests that conformational selection (Di Cera, 2020; Uversky, 2014) plays a critical role in the binding of Prx to ComR, and thus the inhibition of both quorum sensing and natural competence in *Streptococcus pyogenes*.

## 2 RESULTS

### 2.1 Purification and biochemical function of paratox variants

**FIGURE 1:**
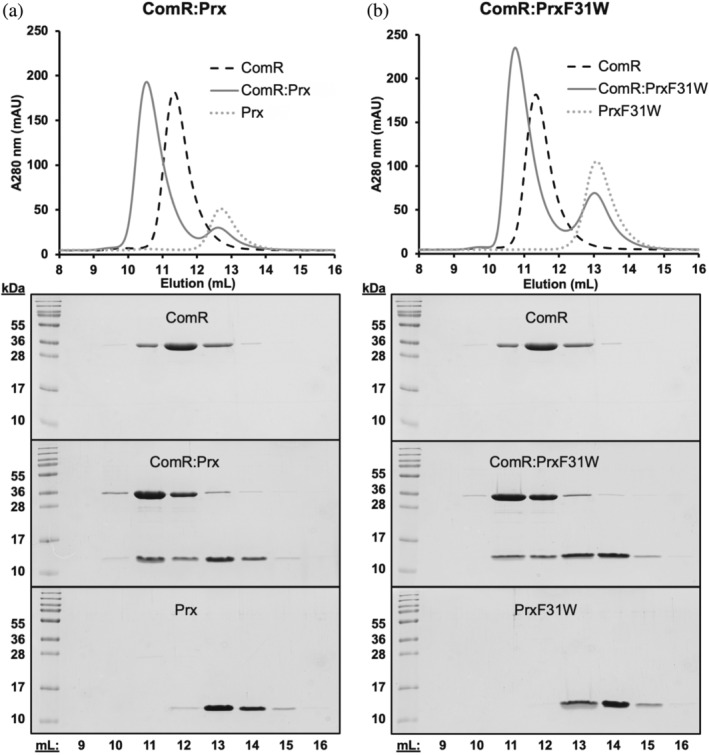
Purification of Prx and a PrxF31W variant. Size exclusion chromatography was performed with ComR:Prx complexes at a 1:1.5 molar ratio with (a) ComR and wild‐type Prx and (b) ComR and PrxF31W. Both wild‐type Prx and PrxF31W have their GST affinity tag removed and lack a 6His‐tag. The upper panels for each show the SEC elution profile of the complexes overlayed over the elution profiles of ComR and Prx or PrxF31W. The lower panels show SDS‐PAGE gels of the SEC eluted proteins or complexes, with each well corresponding to the elution volume of the SEC trace above.

In our previous studies, we observed that the Prx crystal structure (PDBid: 6CKA) predicts a significantly smaller hydrodynamic radius than the size measured by experimental SEC‐SAXS data (Rutbeek et al., 2021). In these studies, we used a Prx expression construct that included a non‐cleavable C‐terminal 6His‐tag (Prx‐6His) (Mashburn‐Warren et al., 2018; Rutbeek et al., 2021). It is not uncommon for cloning artifacts such as a 6His‐tag to affect protein stability (Booth et al., 2018) or even stabilize protein conformations as crystal packing artifacts (Lorente Cobo et al., 2022; Lovering et al., 2011; Shanker et al., 2016). In fact, the Prx‐6His crystal dimer is stabilized by the C‐terminal 6His‐tag of a symmetry mate (Mashburn‐Warren et al., 2018). Given this, we proceeded to create a new Prx expression construct without a 6His‐tag to assay the behavior of Prx in solution. Instead, Prx was cloned with a cleavable N‐terminal GST‐tag. As shown in Figure 1a, the new Prx construct is readily purifiable and binds to its known biological partner ComR.

Additionally, our Prx ortholog of study from Streptococcus strain MGAS315 contains no tryptophan residues, impeding the ability to monitor Prx conformational changes by fluorescence. For example, a fluorescent probe allows one to monitor the effect of solutes on protein folding that would otherwise absorb in the far‐UV range and interfere with techniques such as CD. To address this problem, a point variant in the GST‐Prx construct was made by substituting a tryptophan at phenylalanine residue 31 (PrxF31W). This residue was chosen as it is partially buried in the X‐ray crystal structure (Mashburn‐Warren et al., 2018), making W31 fluorescence a potentially sensitive reporter for the conformation of Prx. The variant PrxF31W was still able to bind ComR, demonstrating that this point mutation did not inhibit the biochemical function of Prx (Figure 1b). It is also important to note that both Prx constructs created here appear to elute from the SEC column earlier than expected based on the predicted hydrodynamic radius from the crystal structure. This agrees with our past results, indicating that the observed crystal structure of Prx does not represent its behavior in solution (Mashburn‐Warren et al., 2018; Rutbeek et al., 2021). For clarity, in this study Prx refers to the wild‐type protein without a 6His‐tag and PrxF31W is the spectroscopic probe variant also lacking a 6His‐tag. Furthermore, material containing a 6His‐tag will be referred to as Prx‐6His.

### 2.2 Structure of Prx in solution

**FIGURE 2:**
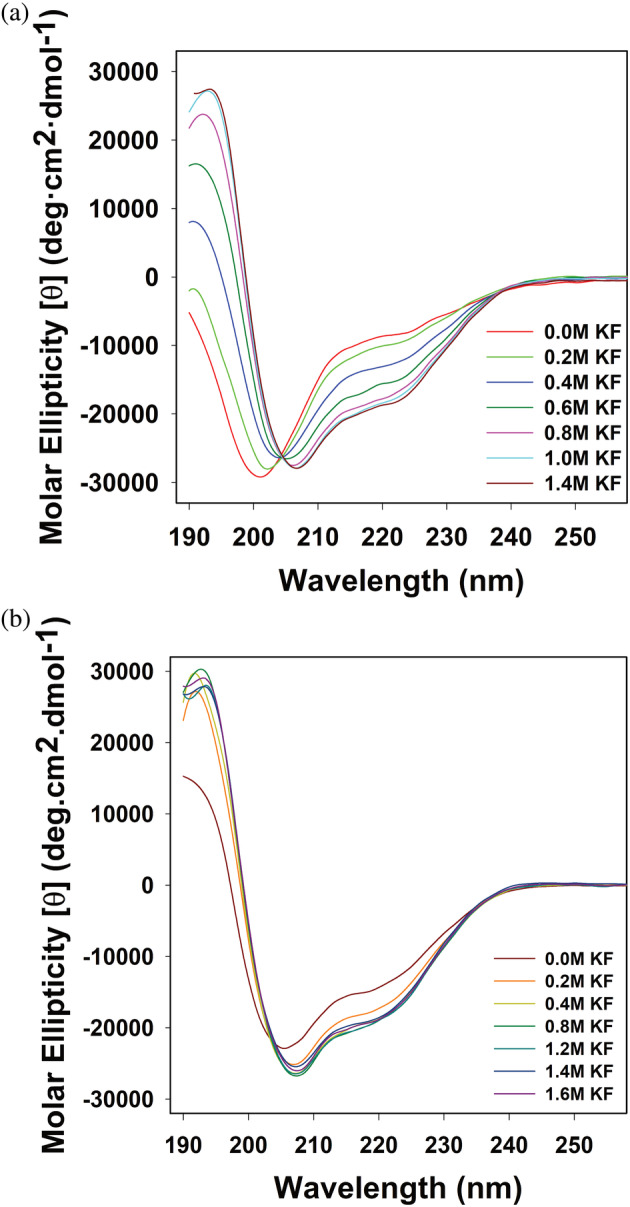
Prx is unfolded in dilute salt conditions. (a) Circular dichroism spectra of Prx secondary structure as perturbed by the addition of potassium fluoride (KF). All measurements were performed in triplicate at room temperature in 20 mM Phosphate buffer (pH 7.0) with a final protein concentration of 20 μM. (b) Circular dichroism spectra of Prx‐6His secondary structure as perturbed by the addition of potassium fluoride. All measurements were performed in triplicate at room temperature in 20 mM Phosphate buffer (pH 7.0) with a final protein concentration of 20 μM.

As our past results suggest that the X‐ray crystal structures of Prx do not properly represent its conformation in solution (Mashburn‐Warren et al., 2018; Rutbeek et al., 2021), we proceeded to assay the structure of Prx in solution. First, we measured the structure of tag‐less Prx in solution by CD. Figure 2a demonstrates how the secondary structure of paratox is affected by salt concentration. It can be clearly seen that under low salt concentrations Prx lacks significant secondary structure, while the addition of potassium fluoride (KF) induces secondary structure in the protein. Overall, this causes Prx to adopt a more compact folded structure. The spectral transition exhibits an isodichroic point around 205 nm, which is consistent with a two‐state model for the folding process (Zwanzig, 1997). We also measured the CD spectrum of the Prx‐6His construct (Figure 2b). Our measurements agree with past CD data (Rutbeek et al., 2021) and indicate that the presence of the poly‐histidine tag induces secondary structure in Prx‐6His.

**FIGURE 3:**
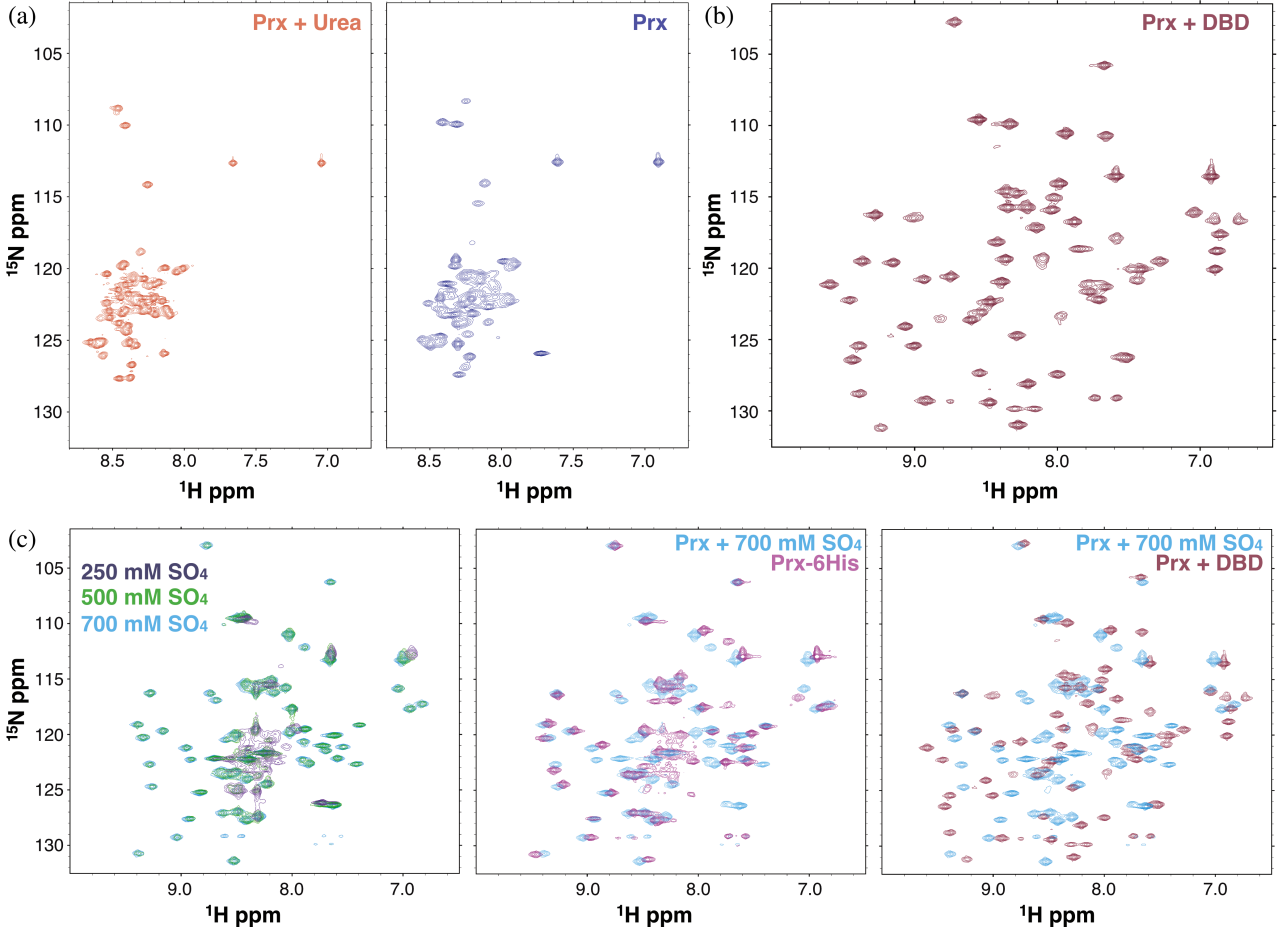
Prx adopts disordered and ordered globular conformations. (a) ^1^H‐^15^N HSQC spectra of tag‐less ^15^N Prx dissolved in 6.66M urea (left) and at pH 7.0 and low salt (right). (b) ^1^H‐^15^N HSQC spectrum of tag‐less ^15^N labeled Prx bound to unlabeled ComR DBD. (c) ^1^H‐^15^N HSQC spectra of ^15^N Prx with increasing Na_2_SO_4_ concentrations (left). Overlay of a ^15^N Prx + 700 mM Na_2_SO_4_ spectrum with a spectrum of ^15^N Prx‐6His containing a C‐terminal 6His‐tag (middle) or with a spectrum of ^15^N labeled Prx bound to unlabeled ComR DBD (right). In each panel the spectra are labeled by color.

As the CD spectrum demonstrated that the structure of Prx was unfolded at physiological buffer conditions, we then used NMR to gain further insight into the solution‐state fold of Prx. Figure 3 depicts several ^1^H‐^15^N HSQC spectra of ^15^N labeled Prx measured in various conditions. Figure 3a shows tag‐less Prx in denaturing conditions (6.66 M Urea) and in buffer at pH 7. It can be clearly seen that in the absence of high‐salt or crowding agents, the ^1^H‐^15^N HSQC dispersion pattern of Prx at pH 7 is similar to that measured under denaturing conditions. Specifically, all backbone chemical shifts show low dispersion and are clustered between 8.0 and 8.6 ppm in the hydrogen dimension. This ^1^H‐^15^N HSQC pattern is diagnostic of a protein that lacks secondary structure and is disordered (Pandey et al., 2023; Prehna et al., 2014). In contrast, the addition of unlabeled ComR DNA‐binding domain (DBD) to ^15^N‐labeled Prx results in a well resolved ^1^H‐^15^N HSQC indicative of an ordered and globular protein fold (Figure 3c). Given the nano‐molar affinity of the Prx:DBD interaction (Rutbeek et al., 2021) and the well‐resolved spectra, we conclude that the HSQC in Figure 3b represents a fold similar to that observed for Prx in the X‐ray crystal structures.

Our CD measurements also showed that the structure of Prx was easily influenced by salt concentration. As such, we have also used NMR to probe the effects of salt upon the structure of Prx (Figure 3c). It can be clearly seen that the addition of sodium sulfate leads to an increase in peak dispersion, inducing an ^1^H‐^15^N HSQC pattern characteristic of a folded protein (Figure 3c, left). Namely, unlike in Figure 3a the peaks are no longer clustered in the center of the spectra which demonstrates that salt has induced the presence of secondary and tertiary structure. If we compare the ^1^H‐^15^N HSQC of tag‐less Prx in 700 mM sodium sulfate to that of Prx‐6His in buffer at pH 7, we see that they are closely related with several overlapping peaks (Figure 3c, middle). Specifically, as a ^1^H‐^15^N HSQC is commonly referred to as the “finger‐print” of a protein, identical HSQC spectra would suggest the same overall fold. This shows that the salt‐induced fold of Prx is similar to the 6His‐tag induced fold of Prx (Prx‐6His) in agreement with our CD data (Figure 2b). However, comparing the ^1^H‐^15^N HSQC spectrum of the salt‐induced folded state with that of DBD‐bound Prx (Figure 3c, right) shows that these Prx structures are not identical. Specifically, we observe that the sodium sulfate Prx spectrum and the spectrum of Prx bound to the DBD have very few overlapping peaks. This is even considering possible differences in Prx chemical shifts due to interaction with the ComR DBD. Taken together, our CD and NMR data demonstrate that in the absence of co‐solutes Prx is an intrinsically disordered protein. The presence of a high concentration of co‐solutes like salts can force Prx to fold, gaining a significant amount of secondary as well as tertiary structure. Additionally, the data shows that Prx ultimately adopts a well‐ordered globular domain when bound to its biological interaction partner ComR. Furthermore, the salt and His‐tag induced Prx folds are similar to each other but distinct from the structure Prx attains when bound to ComR.

### 2.3 Folding properties of paratox

**FIGURE 4:**
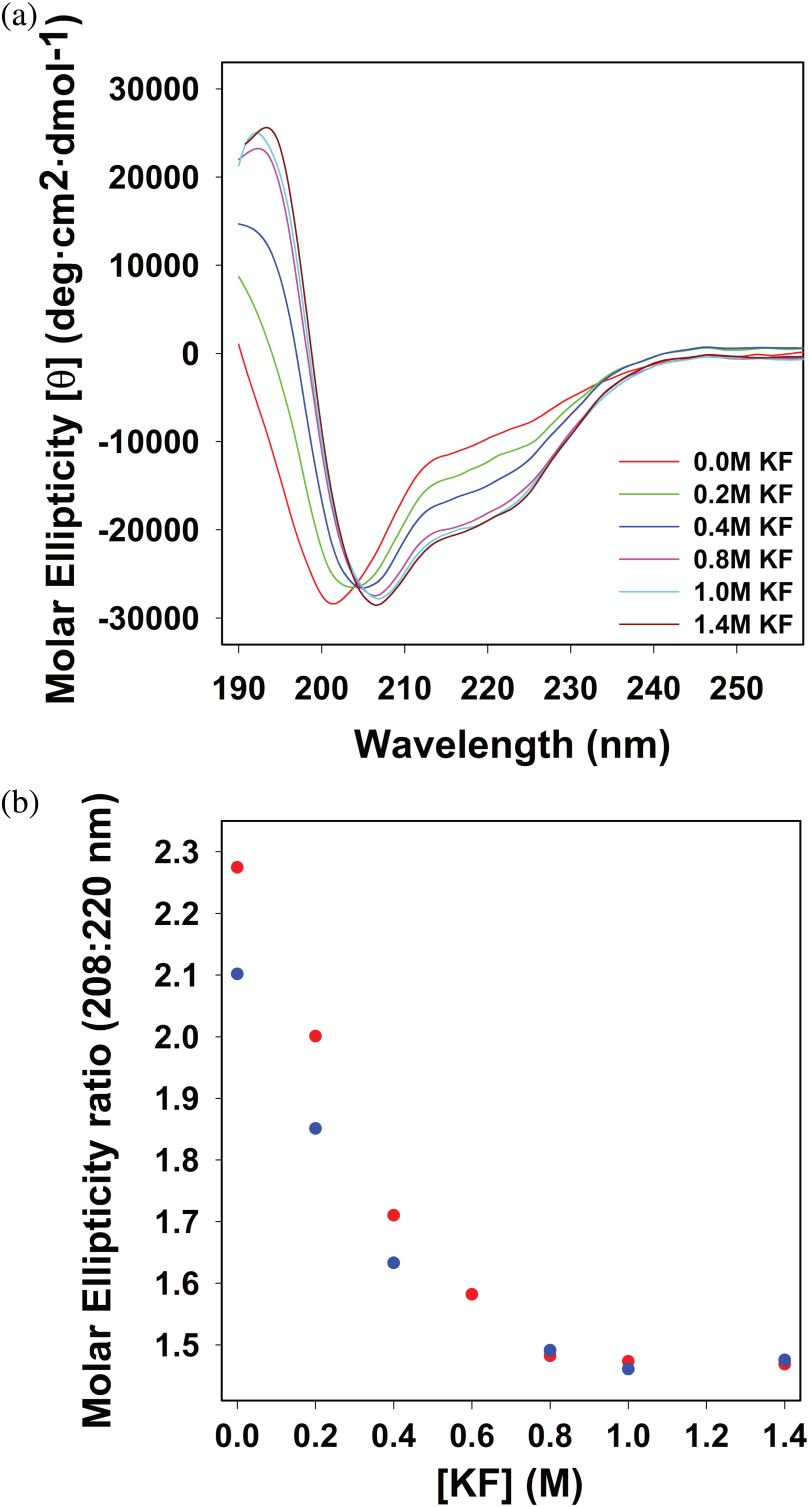
The PrxF31W variant has a comparable folding mechanism to wild‐type Prx. (a) Circular dichroism spectra of PrxF31W secondary structure as perturbed by the addition of potassium fluoride (KF). All measurements were performed in triplicate at room temperature in 20 mM phosphate buffer (pH 7.0) with a final protein concentration of 20 μM. (b) Comparing the KF induced changes in helicity of wild‐type paratox (red) and the paratox F31W variant (blue), as described by the 208:220 molar ellipticity ratios.

Given that Prx is dynamic and can adopt multiple different folds, we used the tryptophan residue of the PrxF31W variant as a spectroscopic reporter to monitor protein folding and dynamics. First, the KF induced folding of the PrxF31W variant was also compared with that of wild type Prx using CD (Figure 4a,b). The CD spectra of Prx and PrxF31W measured at KF concentrations above 1 M are superimposable, indicating that the compact folded forms of wild‐type Prx and PrxF31W have identical secondary structure. Furthermore, the salt‐induced folding profiles of wild‐type Prx and PrxF31W are observed to be very similar, with the minor caveat that the substitution of phenylalanine by tryptophan may have slightly favored the folding of the protein. Interestingly, this is consistent with the small increase in the SEC elution volume of PrxF31W compared to Prx (Figure 1). These results indicate that the F31W mutation only minorly perturbs the thermodynamics of Prx. Considering that the PrxF31W variant does not affect the biochemical function of Prx, our data establishes PrxF31W as an acceptable surrogate for interrogating the folding properties of Prx.

**FIGURE 5:**
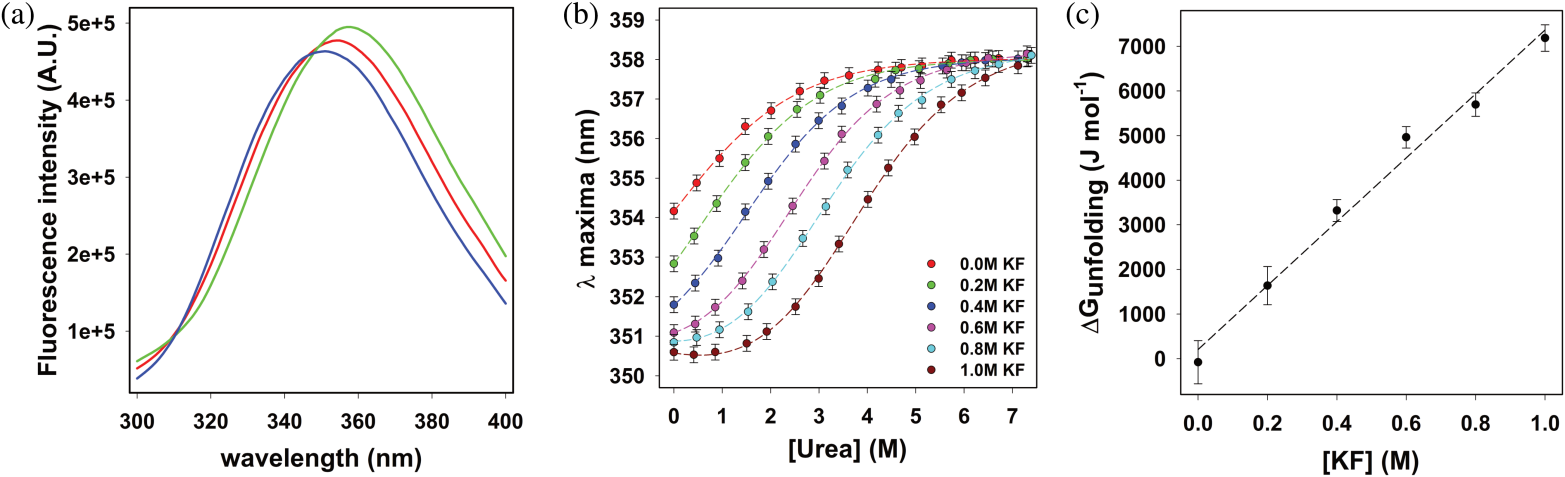
Fluorometric determination of Prx folding states (a) Steady‐state fluorescence spectrum of PrxF31W collected in 25 mM Tris buffer (pH 7.5) buffer (red line), in 25 mM Tris buffer (pH 7.5) containing 1.0M potassium fluoride (KF) (blue line) and in 25 mM Tris buffer (pH 7.5) containing 7.1M urea (green line). All measurements were performed at room 293 K with a final protein concentration of 5 μM. The samples were excited at 280 nm, with excitation and emission slits set to 2 nm bandpass. (b) Urea‐induced unfolding profiles for PrxF31W measured in various KF solutions obtained by monitoring the *λ*_maxima_ of the protein fluorescence peak. All measurements were performed at 293 K, in 25 mM Tris buffer (pH 7.5), with a final protein concentration of 5 μM. The samples were excited at 280 nM and the emission peak was collected from 300 to 450 nm, with both excitation and emission slits set to 2 nM bandpass. The *λ*_maxima_ was obtained by fitting each emission peak to a Weibull 5‐parameter equation (Hallinan, 1993), the error bars representing the uncertainty in *λ*_maxima_ obtained from fitting the data. The solid lines represent the best global fit of the data to Equation (1), sharing the parameters *λ*_U_ (357.6 ± 0.2 nm; the emission maximum of the unfolded protein) and *λ*_F_ (350.2 ± 0.1; the emission maximum of the folded protein). (c) Plotting the dependence of ${∆G}_{\text{unfolding}}^{\left[\text{urea}\right]=0}$ obtained from the data in Table 1 upon KF concentration, the dashed line represents the best linear fit to the data.

Next, the fluorescence properties of PrxF31W were tested in dilute buffer, high‐salt, and denaturing conditions (Figure 5a). When dissolved in pH 7.5 Tris buffer, the protein emission spectrum of PrxF31W exhibits a peak around 354 nm consistent with that of a highly solvent‐exposed tryptophan (Eftink, 1994). The addition of urea causes an additional red‐shift in the protein spectrum, resulting in a fluorescence emission peak around 358 nm. In contrast, the addition of KF causes a blue shift in the protein spectrum, resulting in a measured emission peak around 350 nm. Thus, this data illustrates that the folding/unfolding of PrxF31W between the disordered and the salt‐stabilized form can be probed through monitoring its protein emission peak position. For a protein that unfolds via a two‐state mechanism, the observed maximum emission wavelength of the protein $\lambda_{\mathrm{obs}}$, depends on $x_{F}$ the mole fraction of folded protein: $\lambda_{\mathrm{obs}}=x_{F}\lambda_{F}+\left(1-x_{F}\right)\lambda_{U}$, where $\lambda_{F}$ and $\lambda_{U}$ are the maximum emission wavelengths of the folded and unfolded states of the protein respectively (Monsellier & Bedouelle, 2005). This equation can be used if the quantum yield and fluorescence peak width of the protein do not change significantly as the protein unfolds (Eftink, 1994). This is indeed the case for PrxF31W as can be seen in Figure 5a.

Given our ability to monitor the folding of PrxF31W, urea‐induced denaturation profiles of PrxF31W have been obtained. The folding data is plotted by the protein emission peak maxima as a function of urea concentration and different solvent buffer conditions that contain varying amounts of KF (Figure 5b). As shown, the stability of the folded form of PrxF31W increases with KF addition. This is clearly represented by the urea concentration associated with the folding transition midpoint. From these profiles the standard free energy of folding can be calculated from Equation (1) (Santoro & Bolen, 1992), assuming that PrxF31W folds via a two‐state mechanism:

(1) $$\lambda_{\mathrm{obs}}=\frac{\left(\lambda_{U}^{0}+\alpha_{U}\times \text{[urea]}\right)+\left(\lambda_{F}^{0}+\beta_{F}\times \text{[urea]}\right)\times e^{\frac{\left({∆G}_{\text{unfolding}}^{\text{[urea]}=0}+m_{\text{unfolding}}\times \text{[urea]}\right)}{\mathrm{RT}}}}{1+e^{\frac{\left({∆G}_{\text{unfolding}}^{\text{[urea]}=0}+m_{\text{unfolding}}\times \text{[urea]}\right)}{\mathrm{RT}}}}$$

In this equation, $\lambda_{U}^{0}$ is the emission maximum wavelength of unfolded PrxF31W in a given buffer in the absence of urea, $\lambda_{F}^{0}$ is the emission maximum wavelength of folded PrxF31W in a given buffer in the absence of urea, $\lambda_{\mathrm{obs}}$ is the emission maximum of paratox measured at a given concentration of urea, $\alpha_{U}$ and $\beta_{F}$ linearly correct for the effect of urea upon the spectrum of folded and unfolded PrxF31W, ${∆G}_{\text{unfolding}}^{\left[\text{urea}\right]=0}$ is the standard free energy of unfolding measured in the absence of urea, and$\;m_{\text{unfolding}}$ is the urea denaturation “m‐value” as defined by Pace (Myers et al., 1995). The data in Figure 5b have been fitted to Equation (1), sharing the parameters $\lambda_{U}^{0}$ and $\lambda_{F}^{0}$ for all data sets. The resulting fitting parameters are tabulated in Table 1. The coefficients of determination (*r* ^2^) for all data fits are greater than 0.99, suggesting that the folding of PrxF31W is well‐described by a two‐state model. The “wellness” of these fits suggests that the presence of KF has small effect on the emission properties of folded and unfolded PrxF31W, allowing the maximum emission wavelengths of folded and unfolded PrxF31W in pH 7 Tris buffer (25 mM) to be estimated: $\lambda_{U}^{0}=357.6\pm 0.2$ and $\lambda_{F}^{0}=350.2\pm 0.1$. The contributions of the $\alpha_{U}$ and $\beta_{F}$ are negligible and are not reported.

**TABLE 1:** Unfolding thermodynamic parameters of PrxF31W measured in various KF solutions.

[KF] (M)	$\frac{{∆G}_{\text{unfolding}}^{\left[\text{urea}\right]=0}}{\mathrm{RT}}$	$\frac{m_{\text{unfoldng}}}{\mathrm{RT}}$	*R* ^2^
0.0	−0.03 ± 0.04	−0.8 ± 0.2	0.9988
0.2	0.67 ± 0.12	−0.8 ± 0.1	0.9991
0.4	1.36 ± 0.09	−0.91 ± 0.04	0.9994
0.6	2.30 ± 0.11	−0.89 ± 0.02	0.9997
0.8	2.34 ± 0.11	−0.89 ± 0.02	0.9997
1.0	2.95 ± 0.12	−0.87 ± 0.04	0.9998

*Note*: Parameters were obtained from the best global fit of data from Figure 5b to Equation (1), sharing *λ*_U_ = 357.6 ± 0.2 nm and *λ*_F_ = 350.2 ± 0.1 nm, through non‐linear least square repression analysis.

The standard folding free energy of PrxF31W in non‐crowded dilute conditions (${∆G}_{F}^{0}$) can be determined by plotting the values of ${∆G}_{F}^{\left[\text{urea}\right]=0}$ reported in Table 1 as a function of KF concentration (Figure 5b). As expected, these data are fairly well‐correlated linearly (*r* ^2^ ≈ 0.988) (Figure 5c); allowing for an estimate of: ${∆G}_{\text{unfolding}}^{0}=0.2\pm 0.2\;\mathrm{kJ}/\mathrm{mol}$. PrxF31W is therefore clearly exists in a dynamic equilibrium between the folded and unfolded states under dilute conditions. The absence of any appreciable nonlinear salt concentration dependence of $∆G_{\text{unfolding}}$ within the KF concentration range studied suggests that electrostatic screening plays a minimal role in the salt‐induced folding of PrxF31W. This conclusion is due to the fact that electrostatic screening at salt concentrations between 0 and 1 M should exhibit a pronounced non‐linear effect upon the folding free energy (Francisco et al., 2019; Pegram et al., 2010; Zhang & Cremer, 2009).

**FIGURE 6:**
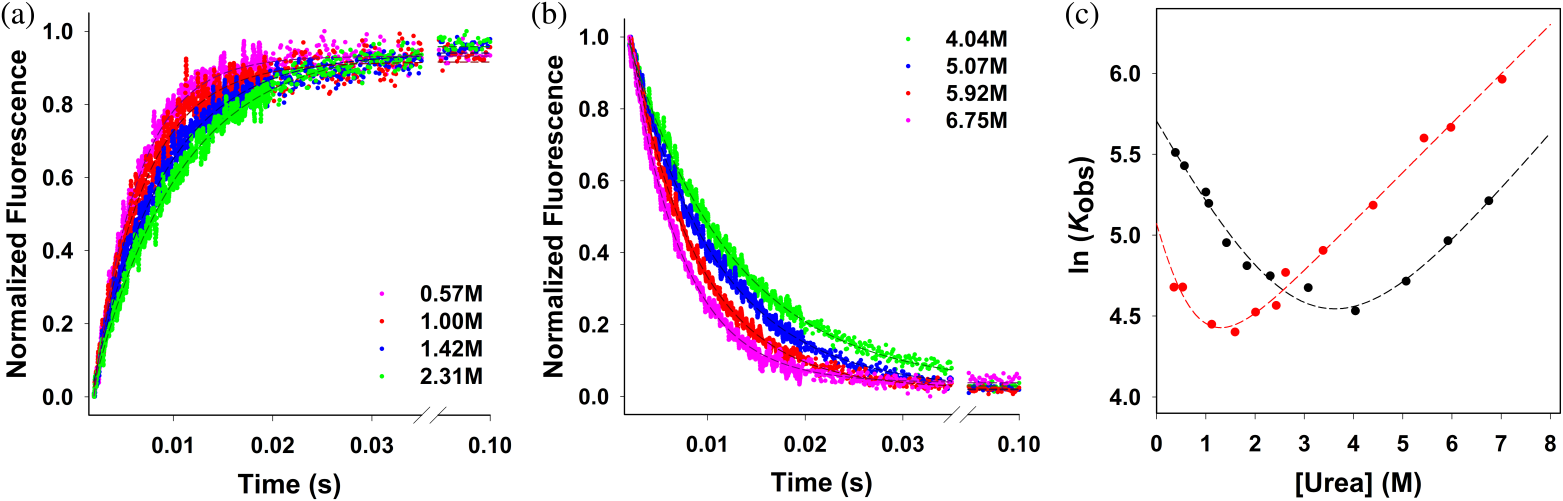
Refolding and unfolding kinetics of PrxF31W shows a two‐state system. Typical stopped‐flow kinetic traces of PrxF31W unfolding and refolding measured in 25 mM Tris buffered at pH 7.5, containing 1.0M potassium fluoride (KF) at 20°C. (a) Prx refolding traces (fluorescence increase) measured in the following urea concentrations are depicted: 0.57M red, 1.00M green, 1.42M blue, 2.31M pink. (b) Prx unfolding traces (fluorescence decrease) measured in the following urea concentrations: 4.04M dark red, 5.07M dark green, 5.92M dark blue, 6.75M dark pink. The change in protein fluorescence is monitored at 330 nm and is normalized to 1 for reasons of clarity. The solid and dashed lines represent the best mono‐exponential fits (Equations (2a) and (2b)) to the obtained data. (c) Chevron plots depicting the observed folding and unfolding relaxation rate constants as a function of urea concentration, shown for 1.0M KF (black) and 0.5M KF (red). All measurements were performed in 25 mM Tris buffered at pH 7.5, fitting errors are smaller than the circle diameters. For each KF concentration, the solid curves represent the best fit of the data to Equation (3).

The salt‐induced folding mechanism of PrxF31W was also studied with stopped‐flow in buffers containing 1 and 0.5M KF to observe the kinetics of PrxF31W transitioning between the unfolded and compact folded states. Lower concentrations of salt were not studied by stopped‐flow because it is impossible to appreciably fold the protein at low salt concentrations. Figure 6 depicts typical stopped‐flow traces of the unfolding/unfolding of PrxF31W in buffers containing varying amounts of urea. Figure 6a represents PrxF31W folding traces and Figure 6b shows PrxP31W unfolding traces. The folding/unfolding fluorescent traces of PrxF31W are well described by single‐exponential kinetics as described by equations:

(2a) $$F=\left(F_{0}-F_{\infty }\right)e^{-k_{\mathrm{obs}}t}+F_{\infty }\left(\text{unfolding}\right)$$

(2b) $$F=F_{0}+\left(F_{\infty }-F_{0}\right)\left(1-e^{-k_{\mathrm{obs}}t}\right)\left(\text{folding}\right)$$

In these equations, t is time, $F_{0}$ is the fluorescence intensity measured at t=0, $F_{\infty }$ is the fluorescence intensity extrapolated to t=∞, and $k_{\mathrm{obs}}$ is the apparent rate constant which for a two‐state folding transition is equal to the sum of $k_{\text{folding}}$ and $k_{\text{unfolding}}$, the folding and unfolding rate constants.

Additionally, we have further investigated the salt‐induced folding of PrxF31W through constructing a chevron plot (Matthews et al., 1983) from our obtained $k_{\mathrm{obs}}$ values as seen in Figure 6c. The dashed line represents the best fit to the equation:

(3) $$\ln k_{\mathrm{obs}}=\ln\left(k_{\text{folding}}^{0}e^{-m\left[\text{urea}\right]}+k_{\text{unfolding}}^{0}e^{-n\left[\text{urea}\right]}\right)$$

In which $k_{\text{folding}}^{0}$ and $k_{\text{unfolding}}^{0}$ represent the folding and unfolding rate constants of the PrxF31W in the absence of urea, while *m* and *n* are constants. The lack of any appreciable rollover observed in the chevron diagrams (Chan & Dill, 1998) and the fact that their minimum points are consistent with the inflection points of the denaturation curves of Figure 5b, strongly suggests that the folding of PrxF31W into the salt‐stabilized fold follows a two‐state mechanism. From the chevron plots the folding and unfolding rate constants in 1M KF ($k_{\text{folding}}^{0,\left[\mathrm{KF}\right]=1M}=285\pm 15\;s^{-1}$ and $k_{\text{unfolding}}^{0,\left[\mathrm{KF}\right]=1M}=16\pm 5\;s^{-1}$) and in 0.5M KF ($k_{\text{folding}}^{0,\left[\mathrm{KF}\right]=0.5M}=112\pm 20\;s^{-1}$ and $k_{\text{unfolding}}^{0,\left[\mathrm{KF}\right]=0.5M}=48\pm 8\;s^{-1}$), can be obtained. If the unfolding free energy of activation is extrapolated to 0M KF, we can estimate the folding and unfolding rate constants in dilute pH 7 buffer to be: $k_{\text{folding}}^{0,\text{dilute}\;\mathrm{pH}\;7\;\text{buffer}}\approx 40\pm 20\;s^{-1}$ and $k_{\text{unfolding}}^{0,\text{dilute}\;\mathrm{pH}\;7\;\text{buffer}}\approx 140\pm 90\;s^{-1}$. Together this indicates that the folded form of PrxFW31 in dilute buffer is dynamic and has lifetime significantly shorter than 10 ms, which is consistent with the disordered ^1^H‐^15^N HSQC spectrum that Prx exhibits in Figure 3. To summarize, our spectroscopic interrogation of the folding/unfolding of PrxF31W matches that of a two‐state protein folding model, which folds and unfolds in the millisecond timescale. This clearly shows that Prx is a highly dynamic protein that rapidly transitions between a compact folded state and an unfolded disordered one.

### 2.4 Salt and crowder effects upon the folding of paratox

**FIGURE 7:**
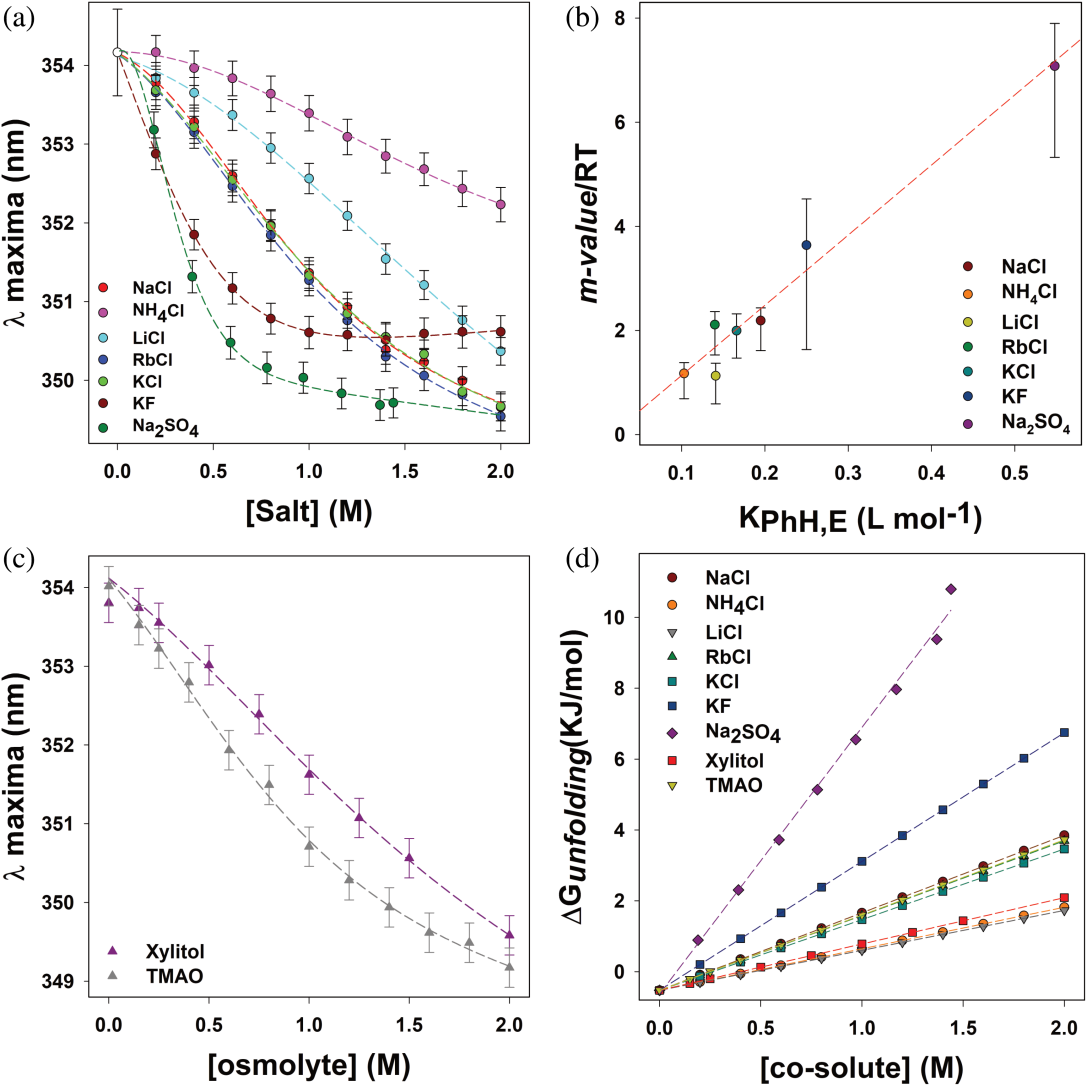
Salt specific effects on the folding thermodynamics of Prx represents hydrophobic collapse. (a) Salt induced effect on the structure of PrxF31W as monitored by the intrinsic tryptophan fluorescence emission peaks (*λ*_maxima_) in various co‐solute solutions. All samples were excited at 280 nM and the emission spectra were collected from 300 to 450 nm, with excitation and emission slits both set to 2 nM bandpass. All measurements were performed in triplicate and averaged, the *λ*_maxima_ was obtained by fitting each emission peak to a Weibull 5‐parameter regression, the error was obtained from the standard deviation of triplicate measurements. The data were globally fit to Equation (4a), constraining *λ*_U_ (the emission maximum of the unfolded protein) to 357.6 nm, while sharing *λ*_F_ (350.5 ± 0.3 nm; the emission maximum of the folded protein) through non‐linear least square regression analysis. (b) Plotting the “m‐value” parameters from Table 2 against the salting out efficiency as represented by the experimentally determined Setschenow constants of benzene obtained from Marcus (2013). The dashed line represents the best linear fit obtained from the data. (c) Osmolyte induced effect on the structure of PrxF31W as described in (a). (d) Dependence of ${∆G}_{\text{unfolding}}^{0}$ of PrxF31W as a function of co‐solute concentration. The data were obtained using the ${∆G}_{F}^{\left[\mathrm{co}-\text{solute}\right]=0}$ and the “*m*‐values” from the global fits of the data depicted in this figure. The dashed lines represent ${∆G}_{\text{unfolding}}^{0}={∆G}_{F}^{\left[\mathrm{co}-\text{solute}\right]=0}+m\left[\mathrm{co}-\text{solute}\right]$ as defined by the parameters tabulated in Table 2.

We have shown that the presence of salts clearly affects the folding of Prx in solution. Furthermore, our PrxF31W unfolding studies with KF demonstrate that the protein unfolding free energy linearly depends upon the salt concentration (Pegram et al., 2010). As such, we proceeded to test the effects of additional salts and crowding agents on the fold of Prx. Figure 7a shows the effects of different salts upon the folding of Prx. Clearly, the salt induced folding of Prx exhibits strong salt‐specificity. This salt‐specificity, coupled to the observed linear dependence of free energy upon salt concentration, strongly suggests that Hofmeister salting‐out effects are perhaps the dominant contributors to the process. Hofmeister effects on the protein folding standard free energy $∆G_{\text{unfolding}}$ are best expressed (Pegram et al., 2010) by the equation: $\frac{\partial ∆G_{\text{unfolding}}}{\partial \left[\text{salt}\right]}=m_{\text{salt}}$. This allows us to modify Equation (1) for this two‐state protein to:

(4a) $$\lambda_{\mathrm{obs}}=\frac{\left(\lambda_{U}^{0}+\alpha_{U}^{\prime }\times \text{[salt]}\right)+\left(\lambda_{F}^{0}+\beta_{F}^{\prime }\times \text{[salt]}\right)\times e^{\frac{\left({∆G}_{\text{unfolding}}^{\text{[salt]}=0}+m_{\text{salt}}\times \text{[salt]}\right)}{\mathrm{RT}}}}{1+e^{\frac{\left({∆G}_{\text{unfolding}}^{\text{[salt]}=0}+m_{\text{salt}}\times \text{[salt]}\right)}{\mathrm{RT}}}}$$

In this equation, $\lambda_{U}^{0}$ is the emission maximum wavelength of unfolded Prx in a given buffer in the absence of salt, $\lambda_{F}^{0}$ is the emission maximum wavelength of folded Prx in a given buffer in the absence of salt, $\alpha_{U}^{\prime }$ and $\beta_{F}^{\prime }$ linearly correct for the effect of salt upon the spectrum of folded and unfolded Prx, ${∆G}_{\text{unfolding}}^{\left[\text{salt}\right]=0}$ is the Prx standard free energy of unfolding measured in pure buffer in the absence of salt and $m_{\text{salt}}$ is the “*m*‐value” characterizing Hofmeister effects as defined by Record (Pegram et al., 2010). The data in Figure 7a have been globally fitted to Equation (4a), sharing the parameter $\lambda_{U}^{0}$ and constraining $\lambda_{F}^{0}$ to be equal to 350.2 nm for all data sets. The resulting fitting parameters “*m*_salt_” and “${∆G}_{\text{unfolding}}^{\left[\text{salt}\right]=0}$”, as well as $\alpha_{U}^{\prime }$ and $\beta_{F}^{\prime }$ are tabulated in Table 2. The salt‐induced folding data of Prx are well‐correlated with Equation (4a) (the coefficients of determination (*r* ^2^) for all data sets are greater than 0.99). Comparing the results of this global fit with the parameters obtained from the urea denaturation results listed above, we notice good agreement in the shared value $\lambda_{U}^{0}=357\pm 2\;\mathrm{nm}$, as well as the values of ${∆G}_{\text{unfolding}}^{\left[\text{salt}\right]=0}$, providing additional confidence in our analysis. The “*m*‐values” depicted in Table 2 demonstrate that each salt affects the protein folding free energy of Prx differently. We can thus rank the efficiency of each salt in promoting the folded state of Prx, based upon *m*_salt_ as:

(5) $${\mathrm{Na}}_{2}{\mathrm{SO}}_{4}>\mathrm{KF}>\text{NaCl}\approx \text{RbCl}\approx \mathrm{KCl}>{\mathrm{NH}}_{4}\mathrm{Cl}\approx \text{LiCl}$$

**TABLE 2:** Salt induced folding parameters of parameters of PrxF31W measured in various saline solutions.

Co‐solute	*m*/*RT*	${∆G}_{\text{unfolding}}^{\left[\mathrm{co}-\text{solute}\right]=0}$ (kJ/mol)	*α*	*β*	*R* ^2^
NaCl	2.2 ± 0.1	−0.6 ± 1	5 ± 4	−0.38 ± 0.04	0.9989
NH_4_Cl	1.2 ± 0.2	−0.6 ± 1	3 ± 2	0.3 ± 0.2	0.9984
LiCl	1.1 ± 0.2	−0.5 ± 1	3 ± 2	−0.7 ± 0.2	0.9986
RbCl	2.1 ± 0.1	−0.6 ± 1	4 ± 4	−0.46 ± 0.04	0.9996
KCl	2.0 ± 0.1	−0.6 ± 1	3 ± 3	−0.42 ± 0.02	0.9984
KF	3.7 ± 0.9	−0.6 ± 1	0 ± 7	0.21 ± 0.02	0.9998
Na_2_SO_4_	7.1 ± 0.2	−0.6 ± 1	26 ± 19	−0.32 ± 0.02	0.9988
Xylitol	1.3 ± 0.2	−0.3 ± 0.5	1 ± 2	−0.8 ± 0.1	0.9990
TMAO	2.1 ± 0.3	‐ 0.3 ± 0.5	2 ± 2	−0.61 ± 0.06	0.9981

*Note*: Parameters were obtained from the best global fit of data from Figure 7a to Equation (4a) and Figure 7c to Equation (4b), sharing *λ*_U_ = 357 ± 2 nm and *λ*_F_ = 350.2 ± 0.1 nm, through non‐linear least square repression analysis.

One concern in applying Equation (4a) is the sensitivity of the obtained *m*_salt_ values towards uncertainty in the $\alpha_{U}^{\prime }$ and $\beta_{F}^{\prime }$ parameters. For the salts studied in this work, both the magnitude and the uncertainty of the $\beta_{F}^{\prime }$ values are small. Therefore, applying this correction has little effect on *m*_salt_ determination. On the other hand, the $\alpha_{U}^{\prime }$ parameters have much larger magnitudes and uncertainties. We have estimated the effect of this uncertainty for each data set in Figure 7a, by fixing all parameters except $\alpha_{U}^{\prime }$ and *m*_salt_ to the values given in Table 2, and then observing how varying $\alpha_{U}^{\prime }$ between its high and low values affects the fit and the resulting *m*_salt_ values. This variation is depicted in the error bars associated with the $m_{\text{salt}}/\mathrm{RT}$ values seen in Figure 7b. It can be clearly seen that although uncertainties in $\alpha_{U}^{\prime }$ may affect the magnitude of *m*_salt_, it does not affect the overall trend given in Equation (5). It must also be pointed out that even though the fitting results shown in Table 2 implies that there is large uncertainty in the $\alpha_{U}^{\prime }$ value associated with KF, our urea denaturation studies indicate minimal KF effects upon the unfolded Prx spectrum. This suggests that $\alpha_{U}^{\prime }$ is small and close to zero, therefore the value of $m_{\text{salt}}/\mathrm{RT}\approx 7.0$ is likely to be close to reality.

Wohl et al. (2021), have shown that salt‐induced conformational changes observed in intrinsically disordered proteins can be caused by salting‐out of the hydrophobic residues, provided that the IDP: (a) is moderately charged (less than 40 percent of sequence residues), (b) has significant hydrophobic content, and (c) has polyampholyte rather than polyelectrolyte character (i.e., positive and negative charges balance out each other). At pH 7, Prx has 28 hydrophobic, 15 negatively charged, and 9–10 positively charged residues out of a total of 60; thus, making it a moderately charged polyampholyte with significant hydrophobic content. Therefore, the salt specific effects we observe in the Prx protein folding free energies can potentially be caused by differences in hydrophobic group salting‐out efficiencies (Francisco et al., 2019; Pegram et al., 2010). How efficiently a salt species reduce the solubility of a given hydrophobic molecule is characterized by the Setschenow constant $k_{\mathrm{Set}}$ defined as (Marcus, 2013):

(6) $$\ln\frac{S_{\text{water}}}{S_{\left[\text{salt}\right]=c}}=k_{\mathrm{Set}}c$$

where $S_{\text{water}}$ is the solubility of the molecule in pure water and $S_{\left[\text{salt}\right]=c}$ is the solubility of the same molecule in a saline solution having a concentration of $\left[\text{salt}\right]=c$. In Figure 7b, we have plotted our measured *m*_salt_ values associated for each salt, compared to its Setschenow constant (Pegram & Record, 2008) for benzene (Marcus, 2013). It can be seen that these values are linearly correlated with each other, confirming that the salt‐induced folding of Prx is essentially caused by the salting‐out of hydrophobic moieties (Francisco et al., 2019).

In addition to salts, osmolytes can also induce Prx to fold as seen in Figure 7c. The data in Figure 7c can be fit to Equation (4b), in which all parameters are defined similar to Equation (4a), with osmolyte concentration being substituted for salt:

(4b) $$\lambda_{\mathrm{obs}}=\frac{\left(\lambda_{U}^{0}+\alpha_{U}^{\prime }\times \text{[osmolyte]}\right)+\left(\lambda_{F}^{0}+\beta_{F}^{\prime }\times \text{[osmolyte]}\right)\times e^{\frac{\left({∆G}_{\text{unfolding}}^{\text{[osmolyte]}=0}+m_{\text{osmolyte}}\times \text{[osmolyte]}\right)}{\mathrm{RT}}}}{1+e^{\frac{\left({∆G}_{\text{unfolding}}^{\text{[osmolyte]}=0}+m_{\text{osmolyte}}\times \text{[osmolyte]}\right)}{\mathrm{RT}}}}$$

The resulting fitting parameters are tabulated in Table *2*. When the concentration dependences of $∆G_{\text{unfolding}}$ upon salt or osmolyte concentration are plotted in Figure 7d, it becomes clear that they all extrapolate within error to essentially identical values of ${∆G}_{\text{unfolding}}^{\left[\text{salt or osmolyte}\right]=0}$. Unlike the salts in Equation (5), which mainly promote protein folding through reducing the solubility of hydrophobic residues, osmolytes stabilize the folded form through reducing the solubility of the polypeptide backbone (Bolen & Rose, 2008; Hu et al., 2010). The fact that the standard Prx unfolding free energy extrapolates to the same value irrespective of perturbing agent, also confirms the fact that the conformational itinerary of Prx can be represented by a two‐state protein folding model.

## 3 DISCUSSION

Under dilute solvent conditions, Prx exists as a disordered and highly dynamic protein. Additionally, at standard physiological buffer conditions Prx remains disordered. For example, both our CD and NMR measurements show that Prx is primarily unfolded in buffers at pH 7.0 and up to 250 mM salt (Figures 2 and 3). Moreover, our titration data strongly suggests that Prx does not adopt a fold that is similar to our X‐ray crystal structures until it binds ComR (Figure 3). The CD and NMR data presented here, in addition to our past AUC (Mashburn‐Warren et al., 2018) and SEC‐SAXS (Rutbeek et al., 2021) data, clearly demonstrate that Prx is an intrinsically disordered protein (IDP) that only adopts a stable globular fold when bound to ComR.

That being said, our studies on Prx and PrxF31W clarify several properties about the structure of Prx in solution. The CD, fluorescence, and NMR results demonstrate that the addition of salt increases both the amount of secondary structure and tertiary structure in the protein. This amount of structural gain is significant enough for us to state that the protein has acquired a fold. We have analyzed our results by postulating that in in solution Prx (and its variants) exist in a two‐state equilibrium between a structureless “unfolded state” and a structured “folded state” having an equilibrium constant $K_{\text{unfolding}}$:

(7) $${\left(\mathrm{Prx}\right)}_{\text{folded}}\overset{K_{\text{unfolding}}}{⇄}{\left(\mathrm{Prx}\right)}_{\text{unfolded}}$$

Analyzing the thermodynamics of Prx via Equation (7) does not imply that Prx only exists in two states. Rather, that this transition can be described by a single structured “folded state” and a large ensemble of unfolded conformations that are at rapid equilibrium with one another (Zwanzig, 1997). Moreover, our experimental thermodynamic and kinetic data demonstrate that promotion of structure in Prx by both salts and osmolytes are adequately described by this two‐state mechanism (Figures 5, 6, 7).

Our measured salt effects upon Prx structure illuminate some interesting aspects about the structure of Prx in solution when analyzed through the prism of Equation (7). Salt effects upon the transition free energy of Equation (7) can be expressed via:

(8) $$∆G_{\left[\text{salt}\right]}^{\text{unfolding}}=∆G_{\left[\text{salt}\right]=0}^{\text{unfolding}}+∆∆G_{\left[\text{salt}\right]}^{\text{electrostatic}}+∆∆G_{\left[\text{salt}\right]}^{\text{hofmeister}}$$

In which $∆G_{\left[\text{salt}\right]}^{\text{unfolding}}$ is the unfolding free energy change of Prx measured in a given salt concentration, $G_{\left[\text{salt}\right]=0}^{\text{unfolding}}$ is the unfolding free energy of Prx measured in the absence of salt, and $∆∆G_{\left[\text{salt}\right]}^{\text{electrostatic}}$ represents the difference between how at a given salt concentration ionic charge screening affects unfolded and folded state free energies. The term $∆∆G_{\left[\text{salt}\right]}^{\text{hofmeister}}$ represents the difference between how salt ions at a given concentration interact with the surface of a protein in its unfolded and folded forms. Additionally, $∆∆G_{\left[\text{salt}\right]}^{\text{hofmeister}}$ is salt specific and always depends linearly upon salt concentration (Francisco et al., 2019; Pegram & Record, 2008; Zhang & Cremer, 2009). It should be noted that a universal equation describing how $∆∆G_{\left[\text{salt}\right]}^{\text{electrostatic}}$ is affected by salt concentration currently eludes the field; this being said, all analyses of $∆∆G_{\left[\text{salt}\right]}^{\text{electrostatic}}$ in the literature confirm that this term should be significantly nonlinear within the salt concentration range of 0–1M (Annunziata et al., 2008; Culham et al., 2016; Record et al., 2013; Rembert et al., 2012; Ren et al., 2012). The fact that $∆G_{\left[\text{salt}\right]}^{\text{unfolding}}$ exhibits only linear dependence upon KF concentration in this range of concentrations for Prx suggests that the contribution of $∆∆G_{\left[\text{salt}\right]}^{\text{electrostatic}}$ must be small. In other words, ionic charge screening is affecting the unfolded and folded state free energies of Prx to the same extent and thus canceling each other.

Interestingly, this result seems to be rather counterintuitive if “unfolded” Prx is assumed to be a long and extended unstructured coil. Especially if we note that Prx is an acidic protein that at both neutral and physiological pH has a significant excess of negative charge. However, a more careful look at the charge distribution of the Prx sequence highlights certain features of the unfolded protein. In the sequence of the Prx ortholog used in our study the fraction of charged residues (FCR) is close to 0.4 and the net charge per residue (NCPR) is close to −0.08. Additionally, the total FCR is comprised of $f_{-}=0.25$ for negatively charged residues and $f_{+}=0.15$ for positively charged residues. According to Pappu's classification of IDP states, this places Prx near the border of the R2 and the R3 conformational classes of IDPs (Das et al., 2015; Holehouse et al., 2017; Mao et al., 2010). In this IDP classification scheme, R2 represents partially compacted IDPs that fold upon binding a target, and R3 includes IDPs that are largely extended but can adopt hair‐pin structures. Additionally, the sequential charge distribution in Prx seems have a noticeable degree of segregation between positive and negative charges. This suggests that “unfolded” Prx can form local compact forms through intra‐chain electrostatic interactions. One can therefore speculate that in the “unfolded” form of Prx, intra‐chain electrostatic interactions have already optimized charge–charge distances for maximum stability and subsequent folding does not change these distances by a large amount. This minimizes the contribution of charge screening to the change in unfolding free energy. Prx therefore only requires a small boost in favorable hydrophobic interactions in order to acquire secondary and tertiary structure, and is thus an R2 IDP. This scenario can also explain the consistency observed between the $∆G_{\left[\text{salt}\right]=0}^{\text{unfolding}}$ values obtained through urea denaturation and the salt and osmolyte titration experiments. Urea unfolds proteins mostly through dispersion interactions rather than electrostatics (Ajayi et al., 2023), therefore $∆∆G_{\left[\text{salt}\right]}^{\text{electrostatic}}$ would be minimally affected by urea addition.

Our work demonstrates that the folded form of Prx observed at high salt concentration is not stabilized through the screening of repulsive charges, but through salting‐out interactions. Our salt and osmolyte data also explain how we were previously able to capture a crystal structure of Prx without a binding partner. Typically, IDPs will not crystallize as the process of crystallization requires a high degree of protein order to form a repeating lattice. However, we had used the purified Prx‐6His construct which we show adopts a similar fold to salt‐stabilized tag‐less Prx (Figure 2 and Figure 3). Also, the crystallization conditions included both salts (0.1M ammonium acetate and 0.1M sodium citrate) and a high concentration of crowding osmolytes (30% PEG 4000) (Mashburn‐Warren et al., 2018). As such, the crystallization conditions and the presence of the 6His‐tag pushed the equilibrium of Prx‐6His towards a folded form. This ultimately caused the protein to crystalize in the globular fold that Prx adopts when bound to the ComR DBD. Our results also strongly advise that when considering the folding and dynamics of any protein, one must consider the effects of His‐tags and other cloning artifacts (Booth et al., 2018).

Given the known X‐ray structures of Prx (Mashburn‐Warren et al., 2018; Rutbeek et al., 2021), the fact that Prx is an IDP (Figures 2 and 3), and our folding analysis (Figures 5, 6, 7), we now suggest that the Prx:ComR binding mechanism may occur through 2 alternative mechanisms involving the following equilibria:

(9a) $$\begin{array}{l} {\mathrm{Prx}}_{\text{unfolded}}\overset{k_{\text{folding}}}{\underset{k_{\text{unfolding}}}{⇄}}{\mathrm{Prx}}_{\text{folded}}\\ {\mathrm{Prx}}_{\text{folded}}+\mathrm{DBR}‐\text{ComR}\overset{k_{\text{binding}}}{\underset{k_{\text{dissociation}}}{⇄}}{\mathrm{Prx}}_{\text{globular}}:\mathrm{DBR}‐\text{ComR} \end{array}$$

Or:

(9b) $$\begin{array}{l} {\mathrm{Prx}}_{\text{unfolded}}\overset{k_{\text{folding}}}{\underset{k_{\text{unfolding}}}{⇄}}{\mathrm{Prx}}_{\text{folded}}\\ {\mathrm{Prx}}_{\text{folded}}+\mathrm{DBR}‐\text{ComR}\overset{k_{\text{binding}}}{\underset{k_{\text{dissociation}}}{⇄}}{\mathrm{Prx}}_{\text{globular}}:\mathrm{DBR}‐\text{ComR} \end{array}$$

In mechanism (9a), Prx is in equilibrium between an unfolded state ${\mathrm{Prx}}_{\text{unfolded}}$, and a folded state ${\mathrm{Prx}}_{\text{folded}}$ as demonstrated by our data. In this mechanism it is essential for Prx to adopt the ${\mathrm{Prx}}_{\text{folded}}$ conformation before interacting with its binding partner. Next, upon encountering ComR, ${\mathrm{Prx}}_{\text{folded}}$ binds the DBD and adopts a final globular fold ${\mathrm{Prx}}_{\text{globular}}:\mathrm{DBP}-\text{ComR}$ through strong favorable interactions with the highly positive DNA binding surface of the ComR DBD (Rutbeek et al., 2021). As ${\mathrm{Prx}}_{\text{folded}}$ is not identical to the ComR bound fold of Prx (${\mathrm{Prx}}_{\text{globular}}$, Figure 3), the binding mode of Prx to ComR (i.e., $k_{\text{binding}}$), also likely includes an induced‐fit component (Arai, 2018; Arai et al., 2015). Overall, this mechanism is a combination of conformational selection and induced fit. In the classical induced fit model described by mechanism (9b), the ${\mathrm{Prx}}_{\text{folded}}$ conformation is an “off the pathway state/conformation” and plays no appreciable role the interaction of Prx with ComR. To fully prove either model will require additional experimentation. For example, studies have shown that IDPs can be forced to switch between conformational selection and induced fit (Arai et al., 2015; Sen & Udgaonkar, 2019), which may allow us to further probe Prx:ComR binding dynamics.

Although we have proposed two possible binding mechanisms for Prx and ComR, our data favors mechanism (9a). First, the fact that osmolyte addition stabilizes the folded form of Prx hints to its putative dominant form *in vivo* before binding ComR. Prokaryotic cells are known to be highly crowded with different solutes, RNA, and protein (Speer et al., 2022). It is therefore likely that Prx is predominantly in the ${\mathrm{Prx}}_{\text{folded}}$ form when it is first expressed in the GAS cell. Second, the fact that Prx is an IDP and conformationally dynamic may help explain the unknown process of how Prx influences the conformation of ComR. Specifically, for Prx to access its binding site on ComR, ComR must undergo an extensive conformational change (Rutbeek et al., 2021; Talagas et al., 2016). The inactive apo‐form of ComR adopts a conformation where the positive DNA binding surface of the DBD is shielded from the solvent and bound tightly to the ComR TPR (tetratricopeptide repeat) domain (Shanker et al., 2016). This conformation prevents ComR from interacting with DNA until XIP binds the TPR to induce a conformational change that releases the DBD (Talagas et al., 2016). Like XIP, Prx also changes the conformation of ComR to release the DBD. However, unlike XIP Prx binds the DNA‐binding residues of the DBD that are shielded by the TPR (Rutbeek et al., 2021). Thus, Prx binding to the apo‐conformation of ComR would result in a large steric clash making it unclear how Prx accesses the DBD. Given that Prx is conformationally flexible and can adopt folds other than what is observed in the crystal structures, it is tempting to speculate that ${\mathrm{Prx}}_{\text{folded}}$ functions as an intermediate that first binds ComR and induces the initial release of the DBD.

Research has shown that IDPs play an increasingly important role in the biology and biochemical pathways of bacteria. For example, the protein Bd0108 is an IDP that regulates pilus secretion and the decision of when to eat other bacteria for *Bdellovibrio bacteriovorus* (Prehna et al., 2014), a triplet of small IDPs was found to affect flagellum gene expression and motility in *Salmonella* (Oguri et al., 2019), and PopZ (polar organizing protein) is an IDP that forms a protein recruitment hub at bacterial poles (Holmes et al., 2016; Nordyke et al., 2020). Our work here now adds Prx to the short but growing list of known bacterial IDPs. Additionally, in eukaryotes it has been accepted that IDPs and protein IDRs (intrinsically disordered regions) form condensates to serve as “membrane‐less organelles” to nucleate protein complex formation and drive biochemical reactions (Uversky, 2017). For example, IDRs in the protein TPX2 create phase‐separation condensates essential for microtubule formation (King & Petry, 2020). Given the prevalence of IDRs and IDPs in bacteria the same condensate hypothesis for IDP biochemical function has been proposed (Cohan & Pappu, 2020). As Prx is an IDP this raises the question if it can also form condensates for a particular biochemical function within *Streptococcus*. This question is especially relevant as small phage proteins are typically evolved to perform multiple functions given the small size of a viral genome (Shah et al., 2021). As we have previously noted that Prx is functionally similar to the multi‐functional phage protein Aqs1 in *Pseudomonas aeruginosa* (Rutbeek et al., 2021), we now hypothesize that the IDP nature of Prx may help facilitate interactions with other yet to be discovered binding partners. Overall, the phage protein paratox is a highly dynamic protein whose fold is readily influence by its local environment and protein binding partners.

## 4 MATERIALS AND METHODS

### 4.1 Protein expression and purification

Expression constructs for Prx included C‐terminal 6His‐tagged Prx (Prx‐6His) and N‐terminal GST‐tagged Prx (Prx). The Prx‐6His construct used was from previous studies (Rutbeek et al., 2021). For the N‐terminal GST‐tagged fusion construct paratox from MGAS315 was placed in the vector pGEX6P1 using BamHI and XhoI sites and ordered from Genscript. For PrxF31W, residue F31 was mutated to W31 using Q5 mutagenesis (New England Biolabs) only in the GST‐tagged construct. Expression constructs for ComR included full‐length ComR and the minimal ComR DNA‐binding domain (DBD) both from *Streptococcus mutans* created in previous studies (Mashburn‐Warren et al., 2018; Rutbeek et al., 2021).

Prx‐6His from MGAS315 was purified as described previously by nickel chelating affinity chromatography followed by size‐exclusion chromatography (SEC) (Mashburn‐Warren et al., 2018; Rutbeek et al., 2021). Both full‐length ComR and the ComR DBD were also purified as previously described using nickel chelating affinity chromatography followed by size‐exclusion chromatography (SEC) (Mashburn‐Warren et al., 2018; Rutbeek et al., 2021). The final buffer in SEC was 50 mM Tris pH 7.5 100 mM NaCl 1 mM beta‐mercaptoethanol (βME).

GST‐tagged Prx proteins were purified as described here. Cells were grown in LB at 37°C to an optical density of 0.8 at 600 nm at which point isopropyl β‐D1‐thiogalactopyranoside (IPTG) was added to 1 mM and the temperature reduced to 20°C. The cells were allowed to grow over‐night and then collected by centrifugation and resuspended in lysis buffer (50 mM Tris pH 7.5 250 mM NaCl 2 mM beta‐mercaptoethanol or βME). Cells were lysed using an Emulsiflex C3 (Avestin) after the addition of 1 mM PMSF, 10 mM MgCl_2_ and DNase I. Lysates were cleared of insoluble debris by centrifugation at 16,000 rpm or 22,000x*g*. The soluble lysate was then passed over a Q‐sepharose gravity column equilibrated in lysis buffer to remove DNA and other contaminants. The flow through was then further purified using a glutathione‐Sepharose 4B (Cytiva) gravity column, washed with 500 mL of lysis buffer and then eluted with lysis buffer containing 50 mM glutathione. The GST‐tag was removed by the addition of HRV‐3C protease with overnight incubation and dialysis into lysis buffer at 4°C. After digestion the sample was passed back over the glutathione‐Sepharose 4B gravity column to remove GST and undigested material. Following GST removal, free Prx was further purified by SEC using a HiLoad 16/600 superdex 75 gel filtration column (Cytiva). The final buffer was 50 mM Tris pH 7.5, 100 mM NaCl, and 1 mM βME. Prx samples were concentrated and flash‐frozen in liquid nitrogen for later use.

For isotopically labeled paratox protein samples, cells were grown in minimal media (M9) but with the nitrogen source substituted to ^15^N ammonium chloride (Cambridge Isotopes). The subsequent steps of cell growth, lysis, and purification are the same as the unlabeled protein samples.

### 4.2 Circular dichroism spectroscopy

Prx, residue point‐variant PrxF31W, and Prx‐6His were dialyzed overnight at 4°C in 1.5 L of CD buffer (20 mM sodium phosphate, pH 7.0) using dialysis membrane with a 2 kDa pore size cut‐off. The final protein concentration was determined using a Thermo Scientific™ NanoDrop™ One Microvolume UV–Vis Spectrophotometer (Prx abs 0.1% = 0.602; PrxF31W abs 0.1% = 1.336). The protein was then diluted to the appropriate stock concentration using CD buffer. A series of 300 μL samples with a final concentration of 20 μM Prx variants were prepared at various concentrations of potassium fluoride. All CD measurement were performed using a Jasco J‐810 spectropolarimeter (Easton, MD). The samples were loaded into a 1 mm pathlength cylindrical cuvette. The spectra were collected in triplicate from 260 to 190 nm for each sample and averaged. Xylitol, TMAO, proline, and urea (ultragrade) were purchased from Sigma Aldrich. All salts and dialysis tubing were purchased from Fisher Scientific (Fair Lawn, NJ).

### 4.3 Nuclear magnetic resonance spectroscopy

^1^H‐^15^N HSQC spectra were recorded at 20°C on a Varian Unity Inova 600 MHz spectrometer equipped with room‐temperature Varian 5 mm Triple‐resonance H/C/N inverse‐detection solution probe with Z gradient probe. Samples were prepared for in their various buffers and included 10% D_2_O. Prx was used at 400 μM concentration in the following buffers all at pH 7.0, 20 mM sodium phosphate, 20 mM sodium phosphate containing 6.66 M urea, 20 mM sodium phosphate containing Na_2_SO_4_ (at 250 mM, 500 mM and 700 mM). For the Prx and ComR DBD complex, 500 μM of unlabeled DBD was added to ^15^N labeled Prx in 20 mM sodium phosphate pH 7.0. ^15^N Prx‐6His samples were measured at 400 μM in pH 7.0 20 mM sodium phosphate buffer. Data was processed using NMRpipe (Delaglio et al., 1995) and analyzed using UCSF Sparky.

### 4.4 Fluorescence spectroscopy

Concentrated PrxF31W was dialyzed in 1 L of buffer (25 mM Tris pH 7.0) for 1.5 h to ensure adequate buffer exchange. The protein was then diluted to the appropriate stock concentration. Steady‐state fluorescence spectra were measured on a Fluorolog‐3 Horiba Jobin Yvon spectrofluorometer (Edison, NJ) using a 10 × 3.3 mm quartz cuvette to hold the sample. The samples were excited at 280 nm, the excitation and emission slits were set to a 2 nm bandpass. All equilibrium folding and unfolding experiments in this work was performed upon protein samples with a final concentration of 5 μM PrxF31W in the buffer of choice. All samples were equilibrated at room temperature (20°C) for 1 h prior to the scan. In the unfolding experiments, Urea stock solutions were prepared for each predetermined concentration of potassium fluoride. The final urea concentrations were determined by refractive index (Grimsley et al., 2006) using 115 V AC/DC Refractometer purchased from Fisher Scientific (Free Lawn, NJ) to verify the final concentration of denaturant post data acquisition. The data were analyzed with Sigma Plot (Point Richmond, CA) software.

### 4.5 Stopped‐flow fluorescence kinetics

Unfolding and refolding kinetics were performed using Applied Photophysics SX‐20 (Surrey, UK) stopped‐flow fluorescence instrument (dead time ~ 1 ms). The excitation wavelength was set to 280 nm, and the emission was monitored using a 330 nm Bandpass filter (FWHM 10 nm). To ensure the appropriate dilution of denaturant, asymmetric mixing was set up using 2.5 and 0.25 mL drive syringes purchased from Delta photonics (Ottawa, CA). In the refolding experiments, concentrated PrxF31W was dissolved in various urea containing potassium phosphate (KF) solutions, and subsequently diluted 10‐fold into the appropriate salt buffer upon mixing. The unfolding experiments were performed in a similar manner whereby, the protein was folded in either 0.5 or 1.0M KF solutions, and then diluted 10‐fold into various urea containing KF solutions. The final protein concentration was between 1 and 2 μM and the final urea concentration was determined by refractive index post mixing. All kinetic experiments were done at 20°C and each measurement was performed at least 15 times and averaged. The kinetic parameters were determined by fitting the data to a mono‐exponential equation using Sigma Plot (Point Richmond, CA) software.

## AUTHOR CONTRIBUTIONS

**Iman Asakereh:** Conceptualization; methodology; investigation; validation; visualization; formal analysis; writing – original draft; writing – review and editing. **Nicole R. Rutbeek:** Methodology; investigation; validation; visualization; writing – original draft. **Manvir Singh:** Investigation; methodology. **David Davidson:** Methodology; investigation; resources. **Gerd Prehna:** Conceptualization; methodology; investigation; validation; funding acquisition; visualization; project administration; supervision; resources; formal analysis; writing – original draft; writing – review and editing. **Mazdak Khajehpour:** Conceptualization; methodology; validation; investigation; funding acquisition; visualization; writing – original draft; writing – review and editing; formal analysis; project administration; supervision; resources.

## FUNDING INFORMATION

This work was supported by the Natural Sciences and Engineering Research Council of Canada (NSERC) RGPIN‐2018‐04968 to G.P., and RGPIN‐2017‐05935 to M.K., and a Canadian Foundation for Innovation award (CFI) 37841 to G.P., and 23175 to M.K.; I.A. was the recipient of a University of Manitoba Graduate Fellowship.

## CONFLICT OF INTEREST STATEMENT

The authors declare that they have no conflicts of interest with the contents of this article.
